# Spatiotemporally precise immune reprogramming: a new paradigm for next-generation neuroimmune therapy in ischemic stroke

**DOI:** 10.3389/fneur.2026.1884472

**Published:** 2026-09-07

**Authors:** Lingling Tuo, Xinyun Li, Jiejin Zhao

**Affiliations:** 1 The First People's Hospital of Xiaoshan District, Hangzhou, Zhejiang, China; 2 Center for Rehabilitation Medicine, Rehabilitation & Sports Medicine Research Institute of Zhejiang Province, Department of Rehabilitation Medicine, Zhejiang Provincial People's Hospital (Affiliated People's Hospital), Hangzhou Medical College, Hangzhou, Zhejiang, China

**Keywords:** biomaterials, immune reprogramming, ischemic stroke, microglia, nanodelivery systems, neuroinflammation, spatiotemporally precise therapy

## Abstract

The neuroimmune response following ischemic stroke is a highly dynamic and complex process with dualistic effects. Traditional broad-spectrum anti-inflammatory strategies are limited in efficacy and may impede repair due to a lack of spatiotemporal specificity. This review systematically proposes and argues for “spatiotemporally precise immune reprogramming” as the core paradigm for next-generation stroke neuroimmune therapy. It critically analyzes the necessity of shifting the paradigm from systemic “immunosuppression” to intralesional “precise reprogramming.” The article further constructs a detailed technical roadmap to achieve this paradigm, encompassing key cellular and molecular targets, advanced delivery systems (such as targeted nanotechnology and biomaterial scaffolds), and the challenges of clinical translation. This review aims to provide a theoretical framework and future directions for developing novel therapeutic strategies that can dynamically modulate the immune microenvironment according to the pathological phase and cellular specificity of stroke to promote neural repair.

## 1 Introduction

Ischemic stroke ranks among the leading global causes of permanent disability and mortality (1). Vascular occlusion triggers a multilayered neuroimmune cascade with dual detrimental and regenerative outcomes, rather than the uniformly harmful inflammatory response once recognized in early research. Resident microglia activate instantly, followed by sequential peripheral infiltration of monocytes, neutrophils and T lymphocytes, jointly sculpting the heterogeneous ischemic microenvironment. This immune response evolves sequentially through hyperacute, acute, subacute and chronic phases with continuously shifting cellular signaling profiles (2). As elaborated in Section 1.2, myeloid immune cells operate along a continuous functional spectrum instead of rigid binary pro−/repair phenotypes, forming the core cellular foundation for targeted immune remodeling. Such spatiotemporal immune heterogeneity renders conventional broad-spectrum anti-inflammatory regimens ineffective, as non-selective suppression inevitably blunts endogenous tissue repair alongside pathological inflammation. Accordingly, therapeutic research must shift from indiscriminate systemic immunosuppression to context-dependent, stage-matched immune reprogramming.

Multiple host-related variables further diversify post-stroke immune trajectories and hinder universal therapeutic design. Age fundamentally reshapes central macrophage-mediated immune trafficking; in aged subjects, central nervous system-associated macrophages drive excessive leukocyte recruitment and aggravated neurological deficits, a phenotype absent in young experimental models (3). Compared with adult animals, juvenile brains mount regenerative, protective inflammatory cascades after ischemia (4). Biological sex also rewires innate and adaptive immune signaling via genetic and chromosomal differences, leading to divergent functional recovery between male and female stroke patients (5). Comorbid conditions including hypertension disrupt vascular adaptive mechanisms and deactivate neuroprotective proteins such as STEP, amplifying post-ischemic inflammatory cascades (6). Even prior mild traumatic brain injury impairs cerebral metabolic and microvascular function, elevating stroke susceptibility (7). Collectively, age, sex, genetics and concurrent illnesses generate highly variable patient immune profiles, making one-size-fits-all anti-inflammatory treatments impractical and highlighting the necessity of individualized precision immunotherapy.

The blood–brain barrier (BBB) represents the primary bottleneck restricting systemic neuroimmunotherapy. As a highly selective homeostatic interface, it blocks over 98% small-molecule agents and nearly all macromolecular biologics from entering brain parenchyma (8). To reach therapeutic concentrations within ischemic lesions, systemic drug administration demands high doses that trigger widespread peripheral side effects and raise infection risk via generalized immunosuppression. Conventional BBB bypass tactics such as osmotic permeabilization and intracranial injection carry invasiveness and off-target tissue damage risks. Though ligand-mediated transcytosis improves endothelial uptake, most peptide-modified carriers still suffer low lesion accumulation efficiency. A broad panel of promising immunomodulatory agents, ranging from natural phytochemicals to pathway-targeted biologics (TREM2, MAPK14), remains preclinically untranslatable due to these delivery bottlenecks. Novel smart biomaterial platforms are therefore urgently required to traverse the BBB, home specifically to ischemic tissue, and release payloads on demand in response to local pathological cues.

Cutting-edge bioengineering and nanomedicine have turned spatiotemporally precise immune reprogramming from theoretical concept into implementable therapeutic logic. Smart biomimetic nanocarriers are designed to resolve the aforementioned BBB and targeting limitations. Nanoparticles coated with platelet or neutrophil membranes naturally accumulate at inflamed cerebral vascular lesions to increase intra-ischemic hemisphere delivery (9). Another design utilizes neutrophil tropism: lipid nanocarriers hitchhike circulating neutrophils to cross the BBB and suppress harmful NET production inside damaged tissue. All such nanosystems are engineered to release payloads only when exposed to lesion-specific signals, including low pH, surplus ROS and high protease concentrations, limiting therapeutic activity exclusively within injured brain tissue. Multi-component nanosystems (layered double hydroxides, polyprodrugs) can co-deliver calcium chelators, antioxidants and anti-inflammatory agents simultaneously to synergistically counteract excitotoxicity, oxidative stress and inflammation. Beyond simple drug delivery vehicles, these integrated carrier technologies establish a versatile engineering toolkit to execute complex stage-specific immune remodeling within the brain’s inflammatory niche.

A wealth of preclinical evidence validates the feasibility of directed immune phenotypic remodeling via engineered delivery systems. hUMSC-derived exosomes suppress TREM1/p38 MAPK inflammatory cascades through HMGB1 cargo to sustain repair-prone microglial states (10). Rosiglitazone-loaded nanoparticles activate PPAR-γ signaling and histone lactylation pathways, simultaneously shifting neutrophils toward N2 protective phenotypes and microglia to repair-biased profiles (11). GLP-1 agonist semaglutide elevates protective Spp1-expressing myeloid populations to mitigate post-stroke neuroinflammation (12). Transient microglial depletion followed by endogenous repopulation also restructures the global immune landscape, restraining peripheral T cell exhaustion and excessive neutrophil differentiation (13). Single-cell and spatial transcriptomic mapping further identifies master regulatory transcription factors (ATF3, STAT6) that govern myeloid state transitions. By integrating high-resolution immune atlas data with lesion-targeted nanodelivery, the field can fully depart from crude systemic immunosuppression and adopt precision immune reprogramming to achieve lasting neurological recovery.

Existing stroke immunotherapies including stage-limited intervention, omics-based personalized modulation and single-target precision strategies are constrained by one-dimensional regulatory logic and oversimplified anti-inflammatory frameworks. Distinct from these established approaches, this review proposes an original spatiotemporally precise immune reprogramming paradigm defined by lesion zone-specific, disease-stage matched intelligent biomaterial intervention. The framework delivers three unique advances: spatially differentiated modulation of infarct core versus penumbra microenvironments, quantitative matching of nanomedicine pharmacokinetics to sequential immune infiltration peaks, and substitution of broad immunosuppression with repair-oriented immune remodeling. This work further constructs a closed-loop translational pipeline spanning immune target identification and smart biomaterial engineering, offering an upgraded theoretical framework for next-generation stroke precision immunotherapy.

## 2 The duality and dynamic evolution of neuroimmune responses: the theoretical basis for a paradigm shift

### 2.1 Phased dynamic changes of post-stroke neuroinflammation and limitations of uniform interventions

The neuroinflammatory response following ischemic stroke exhibits a distinct biphasic temporal evolution, transitioning from an initial pro-inflammatory phase to a subsequent reparative phase, which fundamentally dictates tissue outcome and functional recovery.

In the acute phase, spanning hours to days post-infarction, the immune response is primarily pro-inflammatory, characterized by the rapid activation of resident microglia and the early infiltration of peripheral immune cells such as neutrophils and macrophages (14). This acute inflammatory surge is initially aimed at clearing cellular debris and damage-associated molecular patterns (DAMPs) but, when excessive, exacerbates secondary brain injury by promoting blood–brain barrier (BBB) disruption, oxidative stress, and excitotoxicity (15). Transcriptomic analyses in experimental models reveal a biphasic induction of genes related to DAMPs and pro-inflammatory cytokines, with peaks occurring during the acute and sub-acute phases, underscoring the dynamic and potentially recurrent nature of early inflammation (16). This acute phase is critically driven by metabolic reprogramming, where a glycolytic crisis and lipid peroxidation cascade fuel neuroimmune dysfunction and early deficits (17). The spatial and temporal infiltration patterns of key immune cells, such as macrophages and neutrophils preceding lymphocytes, are comparable between humans and rodents, highlighting conserved pathophysiological mechanisms (18). However, traditional “one-size-fits-all” anti-inflammatory interventions that fail to account for this temporal progression risk therapeutic failure. For instance, premature suppression of all inflammatory activity during the acute phase may inadvertently inhibit necessary clearance processes and early protective signals, while similar interventions during the later repair phase could suppress essential pro-repair mechanisms (19). This is exemplified by studies showing that administering the immunomodulatory neuropeptide cortistatin immediately after stroke was detrimental, whereas delayed administration at 24 h post-stroke was neuroprotective and promoted recovery, emphasizing the critical importance of intervention timing (19).

As the pathology evolves into the sub-acute to chronic phases (days to weeks), the neuroimmune landscape undergoes a crucial shift towards an anti-inflammatory and reparative state. This transition is essential for tissue remodeling, angiogenesis, neurogenesis, and long-term functional recovery (20). During this period, there is a phenotypic switch in key immune cells; microglia and macrophages polarize towards an anti-inflammatory M2 phenotype, which is associated with the release of growth factors and the resolution of inflammation (14). The resolution process is actively governed by the spatiotemporal production of specialized pro-resolving lipid mediators, which counteract the initial inflammatory storm and promote cellular viability (21). Furthermore, chronic phase recovery involves the restoration of metabolic homeostasis, including sirtuin-mediated mitochondrial biogenesis and interactions between key metabolic sensors like AMPK and mTOR, which scaffold the recovery of neural networks (17). The failure of this transition, however, can lead to sustained maladaptive neuroinflammation. Persistent activation of microglia and astrocytes, particularly a shift towards a detrimental A1 astrocyte phenotype in the chronic phase, can drive ongoing neuronal damage and impair recovery (22). This chronic neuroinflammation is linked to long-term complications such as post-stroke cognitive impairment, where sustained glial activation correlates with white matter injury remote from the initial infarct (23).

**Figure 1:**
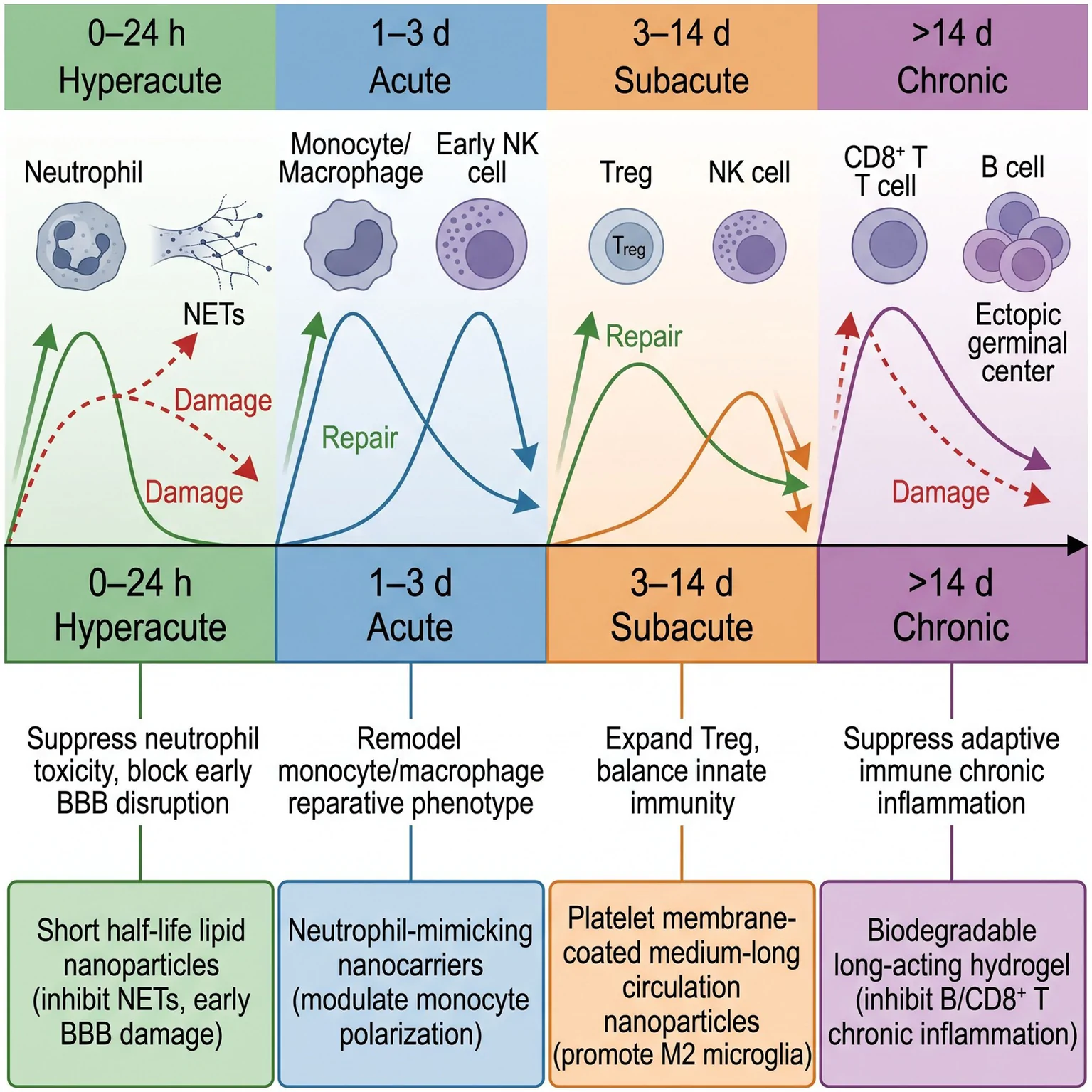
Timeline mapping sequential immune cell infiltration and corresponding spatiotemporal therapeutic windows after ischemic stroke.

Collectively, the simple dichotomy of acute versus chronic inflammation cannot guide precise temporal intervention. The concept of a precise therapeutic “time window,” where interventions must be meticulously aligned with the specific pathological stage, forms the foundational theoretical basis for spatiotemporally precise therapy. Ignoring this dynamism, as seen in clinical trials targeting immune cell invasion that failed due to chronic T-cell proliferation within the brain evading systemic therapy, compromises efficacy and highlights the need for stage-specific strategies (24). Therefore, a deep understanding of the phased immune response—from acute injury mediated by pro-inflammatory cascades to chronic repair driven by resolution pathways—is paramount for designing next-generation therapies that are not merely anti-inflammatory but are intelligently reprogrammed to harness the body’s innate repair mechanisms at the optimal time. To translate this stage-specific therapeutic logic into nanomedicine practice, it is necessary to systematically match the circulation pharmacokinetics of nano-delivery carriers with the sequential infiltration kinetics of immune cell subsets at different disease stages. The sequential infiltration pattern and corresponding optimal therapeutic windows for distinct delivery platforms are visually summarized in Figure 1.

### 2.2 Matching nanomedicine pharmacokinetics with stage-specific immune kinetics

The infiltration curve of immune cells after ischemic stroke follows a fixed chronological order: neutrophils rapidly infiltrate the lesion and reach a peak at 6–24 h post-infarction (hyperacute stage); circulating monocytes infiltrate continuously and macrophage polarization peaks at day 3–7 (subacute stage); B and T lymphocytes gradually accumulate in meninges and peri-infarct tissue from day 14 and persist for months (chronic adaptive inflammatory phase). Different nanomedicine platforms possess drastically different systemic circulation half-lives, which determine their applicable therapeutic windows and targetable immune populations.

First, short-half-life lipid nanoparticles (t₁/₂ < 6 h) are exclusively suitable for the hyperacute neutrophil-dominant window within 12 h after stroke. As reported, lipid nanoparticles loaded with brensocatib hijack circulating neutrophils to cross the BBB and suppress harmful NET formation; their short circulation half-life avoids long-term systemic immunosuppression, yet the rapid plasma clearance restricts sustained regulation of subacute macrophage polarization if administered beyond 24 h (9). Delayed injection of short-half-life nanomedicines fails to intervene in early neutrophil-mediated BBB damage and loses the hyperacute therapeutic window.

Second, medium-half-life biomimetic membrane-coated nanocarriers (platelet/neutrophil membrane camouflage, t₁/₂ = 24–48 h) perfectly match the subacute monocyte/macrophage infiltration peak spanning day 1 to day 3. Platelet membrane nanoparticles carrying deferoxamine maintain stable brain lesion accumulation over 48 h, continuously modulating M1-to-M2 microglia/macrophage phenotypic transformation during the subacute inflammatory peak; their moderate half-life balances targeted lesion retention and low peripheral toxicity (25). However, administration of these medium-half-life carriers in the hyperacute stage will excessively inhibit physiological debris clearance mediated by early innate immune cells, impairing endogenous tissue repair signals (19).

Third, long-acting biodegradable hydrogel scaffolds with degradation cycles of 2–8 weeks are designed for chronic adaptive immune regulation. After injection into the infarct cavity, the scaffold releases immunomodulatory factors (IL-4, BDNF) slowly and continuously, targeting sustained ectopic B lymphocyte follicle formation in late stroke. Conventional injectable nanodrugs with short half-lives cannot reach effective therapeutic concentrations over weeks, so they fail to reverse chronic persistent neuroinflammation (26).

Critical temporal mismatch risks are further summarized: Applying long-duration hydrogel scaffolds at hyperacute phase suppresses necessary early inflammatory clearance; short-half-life lipid nanoparticles cannot alleviate chronic B cell-driven inflammation; medium-half-life biomimetic nanoparticles lose efficacy if administrated outside the 1–3 d subacute macrophage window. Therefore, the core of spatiotemporally precise therapy is to select nanomedicines with matched circulation half-life according to the real-time immune infiltration stage of individual patients, rather than adopting uniform nanocarrier regimens regardless of pathological timing.

### 2.3 Heterogeneity and dual roles of key immune cells

The functional plasticity and phenotypic heterogeneity of key immune cells within the post-stroke brain provide a direct cellular biological rationale for therapeutic “reprogramming” strategies, moving beyond simplistic cell clearance approaches. Microglia and monocyte-derived macrophages act as core orchestrators of post-stroke neuroinflammation. Early research established the simplified binary M1/M2 polarization framework to generalize their opposing inflammatory functions, but recent high-resolution single-cell and spatial transcriptomic profiling has overturned this discrete classification, proving microglial activation forms a continuous, multi-dimensional functional spectrum rather than two mutually exclusive fixed phenotypes (27). Pure M1 or M2 signature marker co-expression is rarely detected in *in vivo* ischemic lesions; numerous transitional, context-specific myeloid subpopulations dominate the dynamic lesion microenvironment instead. Among these newly validated subsets, disease-associated microglia (DAM) represent the most well-characterized pathogenic microglial cluster in stroke tissue, marked by elevated Apoe, Lpl, Cst7, Clec7a transcription. DAM exert stage-dependent dual functions: early DAM enrichment within the necrotic infarct core amplifies TNF-α, IL-1β release and accelerates neuronal pyroptosis, while late DAM remodeling in the metabolically viable penumbra facilitates damaged cellular debris clearance and initiates tissue reconstruction. Beyond DAM, single-cell datasets further resolve multiple specialized microglial subpopulations with distinct spatial distribution across lesions, including interferon-responsive microglia, proliferative microglia and low-inflammatory transitional microglia, each with unique glycolysis/oxidative phosphorylation metabolic signatures that shift dynamically from hyperacute to chronic stroke stages. Similarly, infiltrating monocyte-derived macrophages possess independent transcriptional trajectories distinct from resident microglia and cannot be simply categorized into pro/anti-inflammatory binary groups. Interventions such as rhFGF21 and NBP do not merely drive an artificial M1-to-M2 switch, but tune the continuous functional spectrum of heterogeneous myeloid populations toward a repair-biased transcriptional state, which aligns with the core logic of spatiotemporally precise immune reprogramming proposed in this review. Astrocytes also display phenotypic duality, with detrimental A1 and beneficial A2 states, and their crosstalk with microglia, particularly through pathways like C3/C3aR/NF-κB, is crucial for determining the chronic inflammatory milieu (22). This intricate cell–cell communication underscores that reprogramming must consider the entire neurovascular unit ecosystem.

Neutrophils represent the earliest wave of peripheral immune cells infiltrating ischemic lesions, and early studies uniformly characterized them as pathogenic drivers of acute brain injury. Upon entry within hours of vascular occlusion, neutrophils release reactive oxygen species, matrix metalloproteinases and neutrophil extracellular traps (NETs) to aggravate blood–brain barrier breakdown and secondary thrombosis (28). However, advanced single-cell and spatiotemporal profiling has revised this oversimplified destructive model, revealing context-dependent dual neutrophil functions shaped by disease stage and local microenvironment. Reactive resident microglia can phagocytose infiltrated neutrophils and suppress excessive NETosis as an endogenous homeostatic feedback loop (28). Rather than globally depleting neutrophils—which would abolish essential early debris clearance—therapeutic reprogramming aims to tune their functional phenotype: limiting harmful NET formation in the hyperacute window while preserving mild pro-resolving neutrophil activity in later subacute phases.

Following the early innate immune cascade dominated by myeloid populations, adaptive immune subsets gradually accumulate to govern long-term chronic neuroinflammation, with B lymphocytes serving as key chronic mediators. B cells minimally infiltrate lesions in the hyperacute stage but steadily gather within meninges and peri-infarct tissue from day 14 onward, forming ectopic germinal center-like aggregates that sustain persistent inflammation via autoantibody secretion and pro-inflammatory cytokine release; this pathological B-cell activity correlates with post-stroke tau hyperphosphorylation and permanent cognitive decline (29). Mechanistically, early microglial MIF-CD74/CXCR4 signaling recruits circulating B cells into ischemic parenchyma, and intra-B cell interferon cascades further amplify sustained neurotoxicity (29). Targeted reprogramming strategies therefore seek to disrupt this recruitment axis and restrain aberrant B-cell activation without eliminating protective regulatory B subsets.

Beyond neutrophils, microglia and monocyte/macrophages that orchestrate acute innate inflammation, a spectrum of adaptive and innate lymphoid cells sequentially infiltrate lesions across subacute and chronic stages, jointly shaping the long-term balance between tissue injury and repair, including regulatory T cells (Tregs), cytotoxic CD8^+^ T cells and natural killer (NK) cells.

Regulatory T cells function as the primary endogenous anti-inflammatory lymphocyte subset, with distinct temporal infiltration kinetics: minimal recruitment occurs within the first 5 days, while robust Treg accumulation emerges at day 7 and persists for weeks within peri-infarct and meningeal compartments (4). Tregs secrete IL-10 and TGF-β to dampen overactivated M1 microglia, inhibit cytotoxic T cell proliferation, and suppress detrimental A1 astrocyte polarization, concurrently supporting angiogenesis and neural progenitor survival. Critically, premature massive Treg infiltration during hyperacute ischemia disrupts necessary damage clearance, meaning Treg upregulation must be precisely restricted to repair phases via stage-specific reprogramming.

Cytotoxic CD8^+^ T cells exert predominantly deleterious chronic effects, first detectable in lesions at day 5 and peaking in subacute stages (17). Granzyme and perforin secretion from activated CD8^+^ T cells drive progressive neuronal loss, while their crosstalk with microglia sustains a self-amplifying pro-inflammatory circuit within the penumbra. Unlike Tregs, unregulated CD8^+^ T-cell infiltration exacerbates persistent white matter injury and cognitive dysfunction; therapeutic interventions target their pathogenic effector functions instead of universal pan-T-cell suppression.

NK cells bridge acute innate and subacute adaptive immune signaling, entering lesions within the first 72 h post-stroke with dual-stage activity (15). Early NK-derived IFN-γ amplifies microglial hyperactivation and BBB leakage, while tissue-resident NK subsets remodel the local immune landscape in late recovery by limiting ectopic B-follicle expansion and restraining sustained CD8^+^ T-cell recruitment. Multi-stage reprogramming can modulate NK cell transcriptional trajectories to shift their net function from injury-inducing to tissue-reparative.

The full neuroimmune network cannot be interpreted in isolation of bidirectional central-peripheral immune crosstalk, mediated primarily by meningeal lymphatic vessels and splenic immune remodeling (30). Damaged neurons release DAMPs and tissue antigens that drain through meningeal lymphatics into deep cervical lymph nodes, triggering systemic T, B and NK cell priming; conversely, stroke rapidly induces splenic atrophy and rewires bone marrow immune progenitor output, continuously renewing the pool of circulating immune cells available to infiltrate brain tissue (8). Core signaling axes including MIF-CD74 and systemic cytokine circulation maintain persistent communication between intracranial lesions and peripheral lymphoid organs. This brain-meningeal-spleen circuit dictates that purely intracranial immune modulation cannot achieve sustained resolution of chronic inflammation, and spatiotemporally precise reprogramming must integrate both lesion-targeted delivery and peripheral immune homeostasis regulation.

Across all myeloid and lymphoid populations discussed above, shared functional plasticity forms the core cellular foundation of the proposed reprogramming paradigm. Every immune cell subtype exhibits context-dependent, stage-shifting phenotypes rather than fixed pro- or anti-inflammatory identities. Conventional broad-spectrum immunosuppression indiscriminately blocks both pathological injury signals and endogenous repair machinery; in contrast, spatiotemporally precise immune reprogramming leverages nanomedicine and biomaterial tools to steer cell function in a phase-matched, zone-specific manner, selectively inhibiting harmful subsets while preserving reparative immune activity to transform post-stroke inflammation into a driver of neural recovery.

### 2.4 Fundamental innovation of spatiotemporally precise immune reprogramming paradigm

To explicitly demonstrate the novelty of the core concept proposed in this review, this subsection systematically distinguishes spatiotemporally precise immune reprogramming from three well-documented therapeutic frameworks, and summarizes core dimensional differences in Table 1, followed by an in-depth explanation of three fundamental innovations that advance the research system of ischemic stroke immunotherapy.

**Table 1:** Dimensional comparison between existing immunotherapies and the proposed spatiotemporally precise immune reprogramming.

Evaluation dimension	Stage-specific immune intervention	Personalized immunomodulation	Conventional precision immunotherapy	This work: spatiotemporally precise immune reprogramming
Spatial resolution	Ignore infarct core/penumbra difference; whole-lesion/systemic uniform treatment	Only rely on patient omics stratification, no regional targeting	Single molecular target precision, ignore lesion spatial heterogeneity	Dual spatial identification via pH/ROS/MMP responsive biomaterials to differentiate infarct core and penumbra
Temporal control	Rough acute/chronic binary division, fixed single-stage therapy	Static personalized dosing without matching immune infiltration kinetics	Target locking regardless of dynamic pathological phases	Quantitatively match nanomedicine half-life with sequential immune cell infiltration peaks; multi-stage sequential release design
Core intervention logic	Restrain inflammatory factors; follow simple anti-inflammation thinking	Adjust dosage based on individual baseline immunity	Single-pathway inhibition	Abandon non-selective immunosuppression; actively reprogram immune cells to reparative phenotypes
Delivery technology matching	Independent of smart biomaterials, mainly systemic small molecules	Only biomarker screening, no delivery engineering design	Simple targeted ligands without stimuli-responsive release	Deep integration of multi-stimulus nanocarriers, biomimetic membranes and injectable scaffolds
Theoretical-translation system	Separate pathological mechanism and delivery research	Only clinical stratification theory, lack operable implementation paths	Target screening without spatiotemporal matching roadmap	Complete closed loop: immune spatiotemporal dynamics → target atlas → smart delivery toolbox → personalized clinical regimen

Collectively, the five-dimensional comparison in Table 2 reveals fundamental distinctions between spatiotemporally precise immune reprogramming and three prevailing stroke immunotherapeutic strategies. Conventional stage-specific immune intervention and personalized immunomodulation largely overlook biochemical discrepancies between the infarct core and penumbra within lesioned brain tissue, while conventional precision immunotherapy focuses primarily on individual molecular targets without accounting for regional microenvironmental heterogeneity. Unlike these frameworks, our paradigm leverages pH-, ROS- and MMP-responsive biomaterials to achieve zone-specific drug release, avoiding non-discriminatory immune modulation across necrotic tissue and salvageable penumbral neurons. Existing approaches also adopt rather coarse acute-versus-chronic stratification or static patient-tailored dosing schemes, failing to quantitatively align therapeutic timing with dynamic immune-cell infiltration kinetics; by contrast, the present work establishes a practical matching principle between nanomedicine pharmacokinetics and phase-dependent immune responses across hyperacute, subacute and chronic disease stages. At the mechanistic level, current strategies generally follow an inflammation-suppression-oriented mindset, which may mitigate acute injury yet risk impairing physiological debris clearance and endogenous regenerative programs. Moving beyond broad immunosuppression, our concept advocates directed phenotypic reprogramming of brain-resident and infiltrating immune cells to preserve protective immune functions while dampening pathological inflammation. Furthermore, prior strategies often separate immunological targets from delivery-system engineering: stage-oriented interventions mostly rely on systemic small-molecule administration, personalized immunomodulation emphasizes omics-based patient stratification without actionable delivery tools, and conventional precision immunotherapy employs simple targeting moieties lacking microenvironment-triggered release behavior. Our framework integrates immune-regulatory targets with multi-stimulus nanocarriers and injectable scaffolds, translating immunological concepts into feasible in-vivo intervention modalities. Most importantly, it builds a closed translational workflow spanning spatiotemporal immune-profile characterization, hierarchical target identification, smart-delivery toolbox deployment and stratified therapeutic regimen design, rather than treating pathological mechanisms, drug development and clinical stratification as isolated research modules. Taken together, these contrasts indicate that spatiotemporally precise immune reprogramming is not a marginal refinement of established immunotherapeutic routes, but a re-established two-dimensional (space–time) regulatory system with revised intervention logic and matched bioengineering support.

**Table 2:** Comparative profiles of main immune reprogramming interventions for ischemic stroke.

Intervention category	Representative agents	Immune reprogramming efficiency	Effective duration	Off-target risk	Optimal spatiotemporal therapeutic stage
Small-molecule drugs	Rosiglitazone, MCC950, NBP	Moderate, reversible phenotype regulation	12–72 h	High systemic off-target; mild central non-specificity	Hyperacute & early subacute phase short-term emergency intervention
Biologics (cytokines/antibodies/peptides)	IL-4, anti-PD-L1 mAb, Angiopep-2	High, specific receptor/pathway locking	3–7 d	Low intracranial off-target; high peripheral immune activation risk	Mid-late subacute phase targeted polarization regulation
RNA interference (siRNA/shRNA)	SRSF3 siRNA, NLRP3 shRNA	Very high, permanent transcriptional inhibition	7–14 d	High intracellular transcript silencing off-target risk	Early hyperacute single-pathway blockade
Mesenchymal stem cell-derived EVs	hUMSC-Exos loaded HMGB1	High, multi-pathway synergistic regulation	10–21 d	Extremely low immunogenic off-target	Subacute to chronic long-term tissue repair
Epigenetic modulators	Histone lactylation agonists	Sustained heritable remodeling	>28 d	Moderate genome-wide chromatin non-specificity	Chronic phase persistent immune microenvironment reshaping

## 3 Limitations of traditional anti-inflammatory strategies and the necessity of a paradigm shift

### 3.1 Pharmacokinetic challenges and off-target effects of systemic administration

The systemic administration of anti-inflammatory drugs for ischemic stroke faces formidable pharmacokinetic barriers, primarily due to the restrictive nature of the blood–brain barrier (BBB). This barrier severely limits the brain tissue distribution of most conventional therapeutics, leading to insufficient drug concentrations at the ischemic lesion site (31). Consequently, achieving therapeutic levels within the brain often necessitates high systemic doses, which in turn elevate the risk of hepatotoxicity, nephrotoxicity, and systemic immunosuppression, thereby increasing susceptibility to infections (32). For instance, the clinical translation of promising agents like the iron chelator deferoxamine (DFO) for mitigating ferroptosis in stroke is hampered by its rapid systemic clearance and suboptimal bioavailability when administered systemically (25). Similarly, natural compounds with neuroprotective potential, such as curcumin and quercetin, exhibit poor oral bioavailability and struggle to cross the BBB, limiting their therapeutic application (33, 34). This fundamental delivery challenge underscores the inadequacy of conventional stroke medications, including fibrinolytic agents, which are constrained by short plasma half-lives and a lack of targeted delivery to the ischemic region (31). Beyond mere delivery, systemic immunosuppression poses a significant risk by indiscriminately interfering with both peripheral and central immune surveillance and repair functions. A lack of cellular selectivity means these therapies cannot distinguish between harmful pro-inflammatory immune cells and beneficial subsets involved in repair processes, such as those participating in synaptic pruning or vascular remodeling (19). For example, broad immunosuppression may inadvertently impair the function of regulatory T cells or alternatively activated macrophages that are crucial for resolving inflammation and promoting tissue recovery post-stroke. This non-selective action can disrupt the delicate balance of the neuroimmune axis, potentially exacerbating long-term neurological deficits. The off-target accumulation of systemically administered drugs in peripheral organs further contributes to unwanted side effects and reduces the therapeutic index. These collective limitations highlight the critical need for advanced drug delivery strategies that can overcome the BBB, enhance lesion-specific targeting, and minimize systemic exposure to improve both the efficacy and safety profile of neuroimmunotherapies for ischemic stroke.

### 3.2 Maladaptation to neuroimmune dynamics

**Figure 2:**
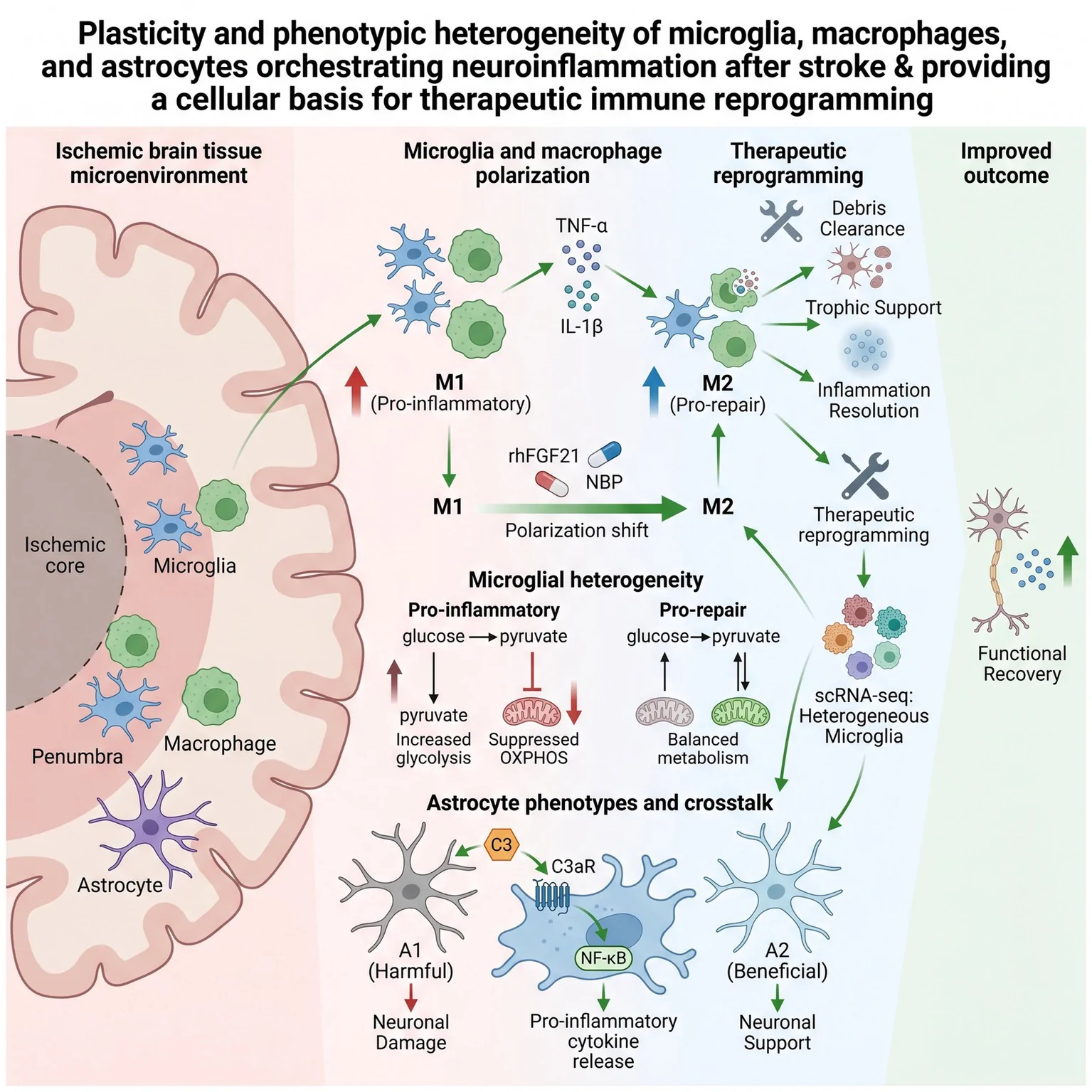
Continuous functional spectrum of microglia/macrophages. Continuous functional spectrum of microglia/macrophages beyond the traditional binary M1/M2 classification, together with dual A1/A2 astrocyte phenotypes that jointly mediate post-stroke neuroinflammation and form the cellular basis of spatiotemporally precise immune reprogramming. Key markers: iNOS (inducible nitric oxide synthase, pro-inflammatory myeloid marker), TNF-α (tumor necrosis factor-α), IL-1β (interleukin-1β); Arg1 (arginase 1), CD206 (anti-repair myeloid marker). Critical signaling axis: C3/C3aR/NF-κB mediates microglia-astrocyte crosstalk driving harmful A1 astrocyte polarization. DAM, disease-associated microglia; OXPHOS, oxidative phosphorylation; scRNA-seq, single-cell RNA sequencing; rhFGF21, recombinant human fibroblast growth factor 21; NBP, dl-3-n-butylphthalide.

Conventional pharmacotherapies for ischemic stroke, often characterized by fixed doses and single molecular targets, are fundamentally maladapted to the highly dynamic and temporally evolving neuroimmune landscape following brain ischemia (Figure 2). The post-stroke inflammatory cascade involves a precisely orchestrated sequence of events where the roles of specific immune cells and inflammatory mediators shift dramatically over time, from early exacerbation of injury to later facilitation of repair. A fixed therapeutic regimen cannot accommodate these phase-dependent changes; for instance, suppressing certain inflammatory signals, such as specific cytokines or microglial activation, during the delayed repair phase may inadvertently hinder essential processes like angiogenesis, neurogenesis, and axonal sprouting (19). This is exemplified by findings that premature administration of immunomodulatory agents at immediate post-stroke time points can be detrimental, potentially interrupting endogenous neuroprotective mechanisms, whereas delayed administration might confer benefit by modulating maladaptive chronic inflammation (19). Furthermore, research underscores that the efficacy of immunomodulatory targets can exhibit significant age-dependent and individual variability. Studies have indicated that responses to certain checkpoint modulators, such as PD-1/PD-L1 axis inhibition, may differ in aged individuals compared to younger subjects, revealing the insufficiency of a “one-size-fits-all” therapeutic approach (19). The neuroimmune response is also influenced by systemic factors and comorbidities, such as periodontitis, which can exacerbate stroke outcome by enhancing systemic and brain inflammation and directly disrupting BBB integrity, thereby altering the local therapeutic milieu (35). These complexities are compounded by the fact that the pathophysiological state itself dynamically alters barrier properties. While stroke-induced BBB disruption can temporarily enhance passive drug extravasation, this window is variable and often accompanied by vasogenic edema and heterogeneous permeability (36). Relying on this passive, pathology-dependent delivery is unreliable. Moreover, the therapeutic window for most agents is narrow, and their pharmacokinetics—such as short half-lives—do not align with the prolonged need for immunomodulation during the subacute and chronic phases of stroke recovery (26). These limitations collectively underscore the critical demand for a new therapeutic paradigm capable of intelligent, spatiotemporal adaptation. An ideal strategy would sense the local disease stage, respond to specific biological cues (e.g., pH, enzymes, or reactive oxygen species at the lesion site), and precisely deliver its payload to specific cellular subpopulations (e.g., pro-inflammatory microglia or infiltrating neutrophils) while sparing reparative immune cells. Such a “smart” therapeutic mode could dynamically align with the neuroimmune timeline, offering phase-specific intervention to inhibit early damage and promote later repair, thereby moving beyond the constraints of static, systemically administered drugs.

## 4 Core target atlas for spatiotemporally precise immune reprogramming

### 4.1 Cellular targets: from innate to adaptive immunity

The precise reprogramming of the neuroimmune landscape in ischemic stroke necessitates a hierarchical targeting strategy across the innate and adaptive immune axes. Microglia and infiltrating macrophages represent the primary and most immediate cellular targets for reprogramming due to their central role as the brain’s resident innate immune sentinels and early responders. Upon activation, these cells can polarize into a spectrum of phenotypes, broadly categorized into pro-inflammatory (M1-like) and anti-inflammatory/reparative (M2-like) states. Promoting a phenotypic switch towards an M2-like state, characterized by the upregulation of markers such as Arginase-1 (Arg1) and CD206 while suppressing M1-associated mediators like inducible nitric oxide synthase (iNOS) and tumor necrosis factor-alpha (TNF-α), is a cornerstone of neuroprotective strategies (37). This reprogramming can be achieved by targeting specific regulatory nodes. For instance, the RNA-binding protein SRSF3 acts as a translational checkpoint, and its targeted knockdown following stroke alleviates translational arrest of innate immune mRNAs in microglia/macrophages, transiently boosting immune signaling and ultimately reducing ischemic lesion size, demonstrating the potential of molecular reprogramming of their activation state (38). Furthermore, targeting specific receptors on these cells, such as peroxisome proliferator-activated receptor-alpha (PPARα) on microglia, can modulate their crosstalk with infiltrating monocytes/macrophages. Ischemia-induced downregulation of microglial PPARα exacerbates inflammation and injury, while its overexpression enhances interleukin-4-mediated communication, promoting a neuroprotective environment (39). Beyond phenotypic markers, targeting specific ion channels or metabolic pathways intrinsic to these cells offers another layer of precision for modulating their function in the ischemic milieu.

Neutrophils, as the first peripheral innate immune cells to infiltrate the ischemic brain, are critical yet double-edged players. Their early, excessive activation contributes to intensive neuroinflammation and worsens outcomes, a process exacerbated by comorbid conditions like hypercholesterolemia, which triggers stronger neutrophil activation and increased formation of neutrophil extracellular traps (NETs) (40). Therefore, reprogramming strategies aim not merely to block their infiltration but to modulate their functional destiny—transforming them from destructive agents into participants in resolution and repair. This can involve engineering drug delivery systems, such as biomimetic nanoparticles, to precisely regulate their infiltration kinetics, inhibit detrimental NETs formation, or even stimulate the release of pro-resolving mediators. The concept of modulating monocyte fate offers a parallel: recruited pro-inflammatory CCR2high monocytes can be cytokine-dependently converted in the brain into CCR2lowCX3CR1high macrophages with reparative functions, highlighting that the adaptation of innate immune cells within the local inflamed milieu is a vital reparative strategy (41). Thus, temporal control is key; early inhibition of destructive neutrophil activities may need to be coupled with later cues to engage their reparative potential.

In the chronic phase following stroke, the adaptive immune system, particularly B lymphocytes, emerges as a pivotal therapeutic target. B cells accumulate in ischemic lesions, forming ectopic germinal center-like structures that correlate with worsened chronic neuroinflammation and injury (29). Their role extends beyond acute damage, potentially linking to long-term complications like post-stroke cognitive impairment and tau pathology. Reprogramming efforts here focus on targeting these ectopic B cell aggregates within the meninges or brain parenchyma. The mechanistic underpinnings involve microglia recruiting B cells via MIF-CD74/CXCR4 signaling early after stroke, while interferon-related pathways within B cells subsequently drive sustained inflammation (29). Therefore, therapeutic strategies could aim to disrupt this recruitment axis, modulate B cell activation states, or regulate their antibody secretion profile to alleviate chronic neuroinflammation. This shift in focus from acute innate responders to chronic adaptive players underscores the importance of spatiotemporal precision in immune reprogramming, where interventions must be tailored not only to the specific cell type but also to the distinct phase of the post-stroke immune response.

### 4.2 Molecular and pathway targets: switches controlling immune cell fate

The functional reprogramming of immune cells in ischemic stroke is ultimately governed by a complex network of molecular switches and signaling pathways. Targeting these upstream regulators offers a powerful strategy to durably alter immune cell fate. Pattern recognition receptors (PRRs) and their downstream inflammasome complexes are critical initiators of the sterile inflammatory response. Sensors like Toll-like receptor 9 (TLR9), absent in melanoma 2 (AIM2), and cyclic GMP-AMP synthase (cGAS) detect abnormal DNA released from ischemic neurons, activating innate immune pathways that can exacerbate injury (42). Among these, the NLR family pyrin domain containing 3 (NLRP3) inflammasome is a central hub, mediating post-ischemic inflammation through interleukin-1β release. Its activation is closely linked to mitochondrial dysfunction, a hallmark of ischemic stroke; damaged mitochondria release contents that can activate NLRP3, creating a vicious cycle of neuroinflammation (43). Therefore, targeting the NLRP3 activation pathway—for instance, by inhibiting upstream signals like RhoA/ASC or mitigating mitochondrial dysfunction—represents a promising approach to suppress excessive inflammasome activation, reduce pyroptosis, and attenuate the inflammatory storm in the acute phase, thereby preserving neural tissue.

Immune checkpoint molecules, such as the programmed cell death protein 1 (PD-1) and its ligand PD-L1 pathway, represent another class of regulatory targets with potential in stroke immunomodulation. However, their application requires careful consideration of timing and context. Emerging evidence suggests that the dynamics of PD-1/PD-L1 expression are not uniform but exhibit location-specific patterns after stroke. For example, in mild ischemic stroke, subcortical infarction is associated with a delayed upregulation of PD-1/PD-L1 regulatory signaling on immune cells compared to cortical infarction, indicating prolonged myeloid-driven inflammation that may be less constrained by checkpoint mechanisms (44). This highlights that the efficacy of checkpoint modulation may be highly dependent on the infarct topology and the individual’s immune state. Broad inhibition or activation may be counterproductive; instead, these pathways may be more applicable to specific patient subgroups or disease stages, such as in modulating chronic adaptive immune responses or in contexts where adaptive immunity is maladaptively engaged, as suggested in post-stroke recrudescence potentially linked to autoimmunity (45).

The functional state and polarization of immune cells are inextricably linked to their metabolic programming. Activated pro-inflammatory cells (e.g., M1 macrophages) typically rely on aerobic glycolysis, while anti-inflammatory/reparative phenotypes (e.g., M2 macrophages) often utilize oxidative phosphorylation and fatty acid oxidation. Reprogramming this metabolic state can indirectly but powerfully drive phenotypic transformation. Activating pathways that promote oxidative metabolism, such as the AMP-activated protein kinase (AMPK) pathway, could potentially shift microglia/macrophages towards a reparative state. This metabolic-immune coupling is a fundamental layer of regulation that extends to trained immunity, where a prior insult (like stroke or infection) leads to long-lasting metabolic and epigenetic reprogramming of innate immune progenitors in the bone marrow, biasing them towards a pro-inflammatory state that can exacerbate secondary injuries or remote organ damage (46). Therefore, metabolic reprogramming strategies aim to reset these maladaptive programs.

For sustained and heritable changes in immune cell function, targeting epigenetic regulators is a frontier approach. Epigenetic mechanisms, including histone modifications and DNA methylation, control the accessibility of genes that define immune cell identity and response. For instance, histone lactylation, a novel epigenetic mark driven by lactate, has been shown to confer an anti-inflammatory gene expression profile in microglia after stroke. Specifically, histone H3 lysine 18 lactylation (H3K18la) regulates the expression of genes like *plxnb2* in microglia, creating a neuroprotective microenvironment (47). Conversely, innate immune memory, which can be detrimental after stroke, is associated with specific histone methylation marks (H3 methylation) that can be mitigated by interventions like mesenchymal stem cell therapy (48). Targeting enzymes responsible for these modifications—such as histone methyltransferases, demethylases, or DNA methyltransferases—offers the potential for durable reprogramming of immune cell gene expression profiles, moving beyond transient signaling blockade to achieve lasting recalibration of the neuroimmune response after ischemic stroke.

## 5 Frontiers in delivery and engineering technologies for achieving spatiotemporally precise reprogramming

### 5.1 Brain-targeted and cell-specific delivery strategies

Achieving precise delivery of therapeutic agents to the ischemic brain and specific cell populations is a cornerstone of next-generation neuroimmunotherapy for stroke. A primary hurdle is the blood–brain barrier (BBB), which, despite becoming more permeable after stroke, still significantly restricts the entry of many drugs and biologics. To overcome this, advanced strategies exploit receptor-mediated transcytosis. For instance, nanoparticles can be functionalized with ligands like the T7 peptide, which targets the transferrin receptor highly expressed on brain endothelial cells, to facilitate transport across the BBB (49). Similarly, the peptide Angiopep-2, which targets the low-density lipoprotein receptor-related protein 1 (LRP1), has been used to modify delivery systems, significantly enhancing brain accumulation of cargoes like neuroprotective agents or gene-editing tools (50). Beyond simple barrier crossing, achieving cell-specific targeting within the complex neuroimmune landscape is critical. This is accomplished by surface functionalization of nanocarriers with antibodies, peptides, or aptamers that recognize unique markers on target immune cells. For example, nanoparticles conjugated with antibodies against vascular cell adhesion molecule-1 (VCAM-1), upregulated on inflamed cerebrovascular endothelial cells post-stroke, have demonstrated remarkable specificity and delivery efficiency to the compromised BBB, achieving nearly two orders of magnitude higher brain accumulation compared to untargeted carriers (51). Aptamers targeting VCAM-1 have also been developed to specifically deliver cargo to cerebrovascular endothelial cells, a key component of the BBB and early responders to ischemia (52). For targeting infiltrating myeloid cells, ligands against surface markers like CD11b (for microglia/macrophages) or Ly6G (for neutrophils) can be employed. A particularly innovative approach leverages biomimetic strategies, such as coating nanoparticles with natural cell membranes. Platelet membrane-coated nanoparticles (Platesomes) exploit the natural homing ability of platelets to sites of vascular injury, conferring excellent lesion-targeting capabilities and immune evasion, as demonstrated for the targeted delivery of the iron chelator deferoxamine to stroke lesions (25). Similarly, neutrophil-like cell membrane coatings can be used to create nanoparticles that actively target the inflamed brain endothelium by mimicking neutrophil chemotaxis (53). Extracellular vesicles (EVs) can also be engineered for dual targeting; for instance, EVs modified with both the RGD peptide (targeting ischemic vasculature) and Angiopep-2 (for BBB penetration) showed enhanced accumulation in the ischemic brain parenchyma (54). Furthermore, rabies virus glycoprotein (RVG)-modified small EVs have been used to achieve neuron-specific delivery of therapeutic peptides (9). These sophisticated brain-targeting and cell-specific strategies collectively aim to maximize therapeutic agent concentration at the desired site of action while minimizing systemic exposure and off-target effects, forming a vital foundation for spatiotemporally precise immune reprogramming.

### 5.2 Stimuli-responsive intelligent drug release systems

The dynamic and pathological microenvironment of the ischemic brain provides unique cues that can be harnessed to design “smart” drug delivery systems for on-demand, spatiotemporally controlled release. Apart from the widely discussed blood–brain barrier transport limitation, ischemic lesions exhibit prominent spatial heterogeneity between necrotic infarct core and metabolically viable penumbra, with distinct pH gradients, reactive oxygen species (ROS) levels and matrix metalloproteinase (MMP) activity in the two regions. These region-specific biochemical signatures serve as the core basis for designing spatially discriminative smart biomaterials. Specifically, the infarct core is characterized by severe acidosis (pH 5.2–6.0), ultrahigh ROS accumulation and abundant MMP-9 secretion, dominated by overactivated M1 microglia and infiltrating neutrophils. In contrast, the penumbra presents mild weak acidification (pH 6.5–7.0), moderate ROS concentration and low MMP-2 expression, containing mixed M1/M2 microglia and surviving functional neurons. Single-stimulus nanocarriers cannot distinguish such divergent regional cues, leading to non-specific off-target drug leakage, while multi-stimulus cascade-responsive nanoplatforms enable precise identification of zone-specific signals to realize spatially selective payload release.

Microenvironment-responsive nanosystems are engineered to remain stable during circulation but undergo structural changes or degradation upon encountering specific lesion signals to trigger localized cargo release. Three core pathological signals in ischemic tissue (pH shift, excess ROS, elevated MMPs) have been exploited to fabricate single-stimulus responsive carriers, each with unique spatial targeting preference matching core or penumbra microenvironment.

First, pH-responsive boronic ester-modified liposomes take advantage of severe acidosis inside the infarct core. Acid-labile boronic linkages only cleave at pH 5.2–6.0 to disassemble liposomes and release NLRP3 inhibitor MCC950, suppressing pyroptosis in necrotic tissue without interfering reparative M2 microglia in mildly acidic penumbra. In contrast, Angiopep-2 peptide-conjugated liposomes lack acid-sensitive groups and selectively bind penumbral vascular endothelium, achieving regionally differentiated NLRP3 inflammasome inhibition across heterogeneous lesions (55). General boronic ester-based pH nanomaterials can also be loaded with rapamycin to modulate microglial polarization and neurovascular repair within acidic ischemic tissue (55).

Second, ROS-responsive thioketal-containing polymers are developed to target the moderate-to-high ROS microenvironment of the penumbra. The representative poly (2,2′-thiodiethylene 3,3′-thiodipropionate) (PTT) nanoparticles degrade selectively under penumbra-specific ultrahigh ROS, functioning as both built-in antioxidants and carriers for PPAR-γ agonist rosiglitazone (56). Pure thioketal backbones barely respond to low-level ROS in chronic peri-infarct areas; composite grafting of phenylboronic ester groups is required to enable dual responsiveness to both acute high ROS and sustained mild ROS gradients (47, 56).

Third, MMP-cleavable peptide-conjugated nanocarriers distinguish lesion zones by differential MMP subtypes: they only break down under low-abundance MMP-2 restricted to the penumbra, releasing therapeutic agents to protect surviving neurons, while remaining intact against high MMP-9 concentrations in the necrotic infarct core to avoid unnecessary drug leakage (57).

To further elaborate the inherent linkage between nanomaterial chemical structure and intracellular immune signaling cascades (rather than simply listing carrier types), we dissect two classic responsive nanoplatforms with full molecular cascades and critical translational evaluation based on the above pH and ROS responsive frameworks.

#### 5.2.1 Thioketal-linked PTT polymer nanoparticles (penumbra-targeted ROS-activated system)

The thioketal moiety embedded in PTT skeletons stays stable under systemic physiological low-ROS circulation and only degrades within the ROS-enriched penumbra to release rosiglitazone. The complete downstream immune reprogramming cascade is as follows: activated PPAR-γ translocates into microglial nuclei to repress NF-κB transcriptional activity, meanwhile upregulating histone lactylation transferase to boost histone H3 lysine 18 lactylation (H3K18la). This epigenetic modification activates reparative genes such as Plxnb2 and drives sustained M2 microglial polarization; concurrently, rosiglitazone blocks the neutrophil NOX2 axis to curb toxic neutrophil extracellular trap (NET) assembly and secretion (47). Critically, single thioketal polymers show negligible degradation under chronic mild peri-infarct ROS, limiting late-stage therapeutic efficacy. Composite phenylboronic ester modification is proposed to extend the ROS response spectrum and prolong the immune reprogramming therapeutic window (54). All structural-signaling correlations of PTT nanoparticles are validated by published mechanistic studies (47, 56).

#### 5.2.2 Boronic ester-grafted MCC950 liposomes (infarct core-targeted pH-activated system)

These liposomes rely on core-specific severe acidosis to cleave boronic ester bonds and liberate MCC950. Free MCC950 directly binds the NACHT domain of NLRP3, blocks ASC oligomerization and downstream caspase-1 activation, and terminates pyroptotic IL-1β secretion from necrotic neurons and hyperactivated resident microglia. A key structural drawback of single boronic ester liposomes is non-specific cargo leakage under mild tissue acidosis, which causes peripheral off-target immunosuppression. Coating an outer MMP-responsive peptide layer forms a multi-stimulus sequential release structure that confines drug release strictly within the infarct core. Cross-comparison with Angiopep-2 vascular-targeted liposomes further demonstrates how surface chemical decoration dictates spatial tropism and region-specific inflammasome suppression.

Beyond single-stimulus carriers, multi-layered sequential release nanosystems and composite hydrogels are designed to simultaneously match the spatial heterogeneity and temporal dynamic evolution of post-stroke neuroinflammation. These chronotherapeutic platforms integrate dual-stage degradation kinetics: the outer shell responds to infarct core high ROS signals to release acute anti-inflammatory cargos (IL-10 mRNA, dexamethasone) and alleviate early necrotic injury, while the inner core decomposes slowly in response to penumbral mild MMP activity to continuously secrete pro-repair factors (BDNF, VEGF) that facilitate angiogenesis and neurogenesis over subacute phases (51).

Temporal control can also be realized via cell-homing nanoparticle formulations tailored to distinct immune infiltration windows. For example, brensocatib-loaded neutrophil-targeted nanoparticles intervene in the hyperacute neutrophil-dominant phase to suppress NET-mediated neurotoxicity (9). Intranasal thermosensitive in-situ gels offer another long-acting delivery strategy, sustaining therapeutic brain concentrations over extended recovery periods (58).

**Figure 3:**
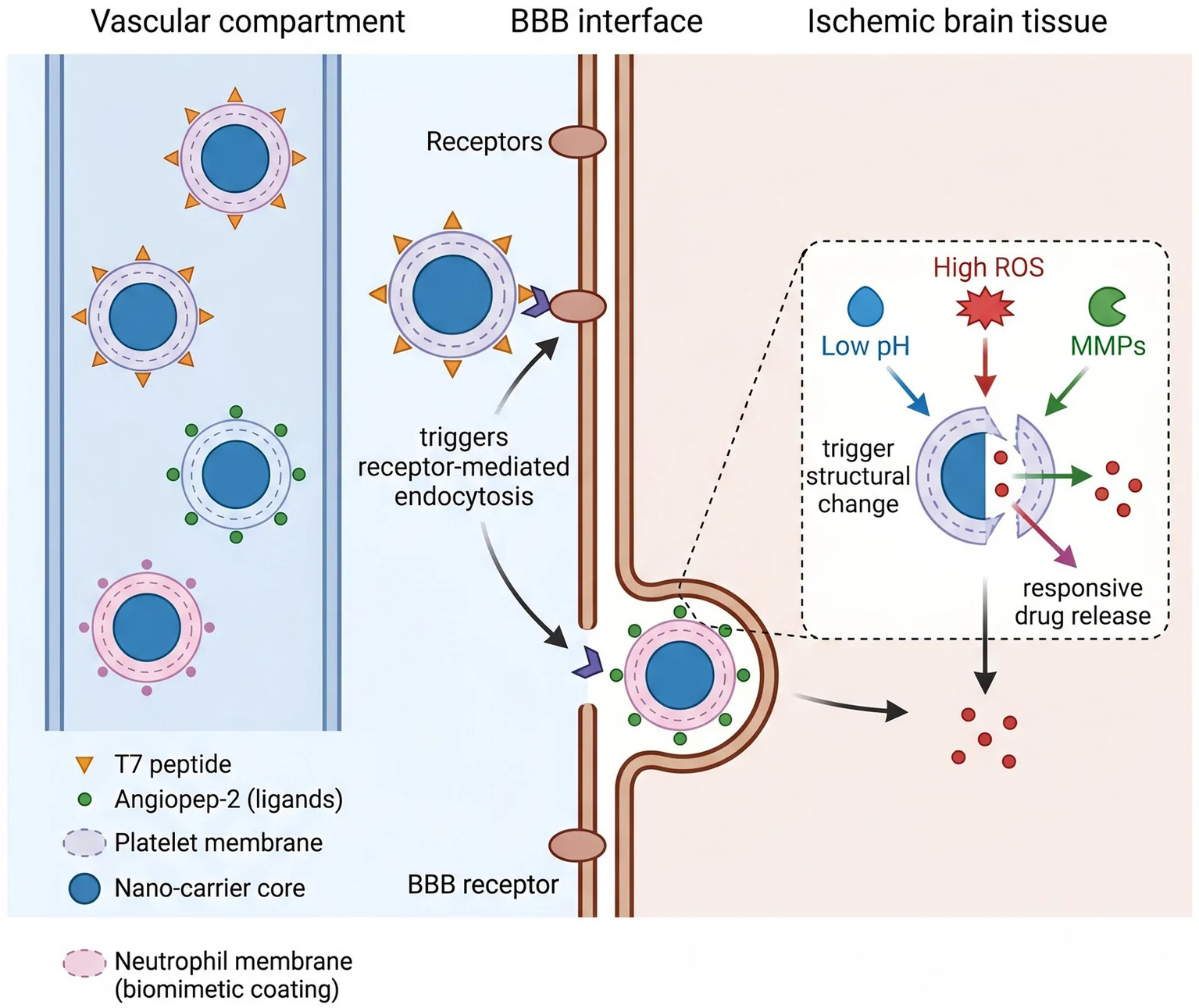
Schematic of smart nano-platforms. Schematic diagram of multi-targeted smart nanoplatforms for blood–brain barrier (BBB penetration and lesion-specific drug release). Nanocarriers are surface-functionalized with T7 peptide, Angiopep-2, platelet membrane or neutrophil biomimetic coating to realize receptor-mediated transcytosis across brain endothelial cells. After entering ischemic tissue, low pH, high ROS and elevated MMPs trigger structural disassembly of nanovehicles to release immunomodulatory cargo locally. ROS, reactive oxygen species; MMP, matrix metalloproteinase; LRP1, low-density lipoprotein receptor-related protein 1.

This section abandons superficial enumeration of biomaterials and targets, and instead provides systematic critical assessment of each delivery system’s advantages, inherent defects and feasible structural optimization strategies for clinical translation. Collectively, these intelligent, pathology-responsive nanoplatforms mark a paradigm shift from passive systemic diffusion to lesion-specific on-demand release, substantially boosting therapeutic precision and reducing systemic adverse effects in ischemic stroke treatment (Figure 3).

To further interpret the differences between intervention modalities listed in Table 1 from the perspective of spatiotemporally precise therapy, we conduct a detailed horizontal comparative analysis focusing on the three core modalities raised by the reviewer (small molecule drugs, biologics, RNAi). Small-molecule compounds are easy to prepare and systemically administer, yet their reprogramming efficiency is moderate and effects are reversible; their short effective duration (12–72 h) can only match the hyperacute neutrophil infiltration window, accompanied by prominent systemic off-target side effects, which necessitates BBB-penetrating smart nanocarriers to improve lesion enrichment. Biologics exhibit superior target specificity with negligible intracranial off-target effects, realizing high-efficiency immune pathway locking and sustained regulation for 3–7 days, perfectly fitting the subacute monocyte/macrophage polarization stage; however, large molecular weight severely limits their natural blood–brain barrier permeability, and peripheral immune activation risks restrict unmodified *in vivo* application. RNA interference achieves the strongest transcriptional-level immune reprogramming with an effective window of up to 14 days, suitable for single-pathway blockade in the ultra-early phase, yet its prominent intracellular homologous transcript silencing off-target risk may trigger unexpected neurotoxicity without cell-specific targeted delivery.

As supplementary therapeutic modalities, mesenchymal stem cell-derived extracellular vesicles balance high synergistic reprogramming efficiency and minimal off-target hazards, serving as the optimal long-term intervention option spanning subacute and chronic phases; epigenetic modulators provide permanent phenotypic remodeling but carry moderate genome-wide non-specific chromatin disturbance risks, which are only applicable to late chronic neuroinflammation control when delivered via localized biomaterial scaffolds. Combined with the spatial heterogeneity of infarct core/penumbra and nanomedicine half-life matching rules elaborated above, a staged combined intervention strategy can be formulated: short-acting small-molecule nanomedicines for hyperacute injury suppression, nanomodified biologics or EVs for subacute M2 microglia polarization, and epigenetic regulator-loaded hydrogels to resolve chronic persistent inflammation.

#### 5.2.3 Engineered extracellular vesicle delivery platforms

Extracellular vesicles (EVs, mainly small exosome-sized vesicles) represent a category of natural cell-derived nanocarriers with inherent advantages over synthetic polymer/lipid nanoparticles for central nervous system intervention, including superior biocompatibility, low intrinsic immunogenicity, and native capacity to traverse the intact blood–brain barrier via endogenous transcytosis pathways (10). Unmodified native EVs secreted by mesenchymal stem cells, neural progenitor cells or endothelial cells carry endogenous anti-inflammatory miRNAs, cytokines and metabolic regulators; systemic administration of unengineered stem cell EVs can moderately shift microglia toward reparative transcriptional states and reduce NET-mediated acute brain injury (10). Nevertheless, natural EVs suffer critical drawbacks: unrestricted systemic biodistribution, short circulation half-life (<6 h), and non-specific uptake by hepatic and splenic reticuloendothelial organs, leading to negligible enrichment within infarct core or penumbra lesions (11). To resolve these limitations, multiple surface engineering strategies have been developed to equip EVs with spatiotemporal targeting capability matching stroke pathological gradients.

Major EV surface engineering approaches for ischemic stroke rely on membrane peptide functionalization to realize dual BBB penetration and lesion tropism. Angiopep-2 conjugated onto EV membrane Lamp2b proteins enables high-affinity binding to brain endothelial LRP1 receptors, boosting trans-BBB transport efficiency (54). Dual modification combining Angiopep-2 and RGD peptide further confers specific adhesion to integrin αvβ3 overexpressed on damaged penumbral vasculature, creating EVs that simultaneously cross barriers and accumulate in salvageable peri-infarct tissue (11). Rabies virus glycoprotein (RVG) decoration offers an alternative neuron-targeting route, while neutrophil-mimetic EV membrane coating recapitulates leukocyte chemotaxis to inflamed ischemic endothelium without triggering peripheral immune activation (53). Beyond surface targeting, cargo engineering via electroporation, transfection or co-incubation allows loading of small-molecule NLRP3 inhibitors, PPARγ agonists, siRNA against pro-inflammatory transcripts or reparative growth factors inside EV lumens, enabling multi-pathway immune modulation once vesicles are released within the lesion microenvironment (4). Combined surface-cargo engineered EVs integrate spatial lesion discrimination and stage-matched payload release, fitting the core logic of spatiotemporally precise immune reprogramming.

Mechanistically, engineered EVs exert multi-tiered neuroimmune regulatory effects spanning hyperacute to chronic stroke phases. In the hyperacute window, EV-carried antioxidants and NETosis suppressors limit neutrophil-mediated BBB breakdown and pyroptosis in the necrotic infarct core. During subacute recovery, encapsulated epigenetic modulators such as lactate precursors drive histone lactylation (H3K18la) to sustain M2-like microglial polarization, clearing cellular debris and stimulating angiogenesis (47). For long-term chronic inflammation, EV-delivered anti-interferon signals restrain ectopic B follicle formation and limit cytotoxic CD8^+^ T cell infiltration in meningeal and peri-infarct regions. Unlike single-target synthetic nanomedicines, EVs deliver synergistic multi-component cargoes to simultaneously remodel the full spectrum of innate and adaptive immune cell subsets, avoiding the single-pathway bias of small-molecule interventions.

Despite their unique biological merits, EV therapeutics face a series of unresolved translational hurdles that restrict bench-to-bedside progression, which were absent from the original manuscript’s brief mention. First, industrial scalable production remains a major bottleneck: conventional cell culture yields extremely low EV concentrations, and large-batch 3D bioreactor culture systems with standardized serum-free media are still under optimization to cut manufacturing costs and ensure batch consistency. Second, inherent vesicle heterogeneity originating from variable parent cell metabolic states generates inconsistent cargo profiles between EV batches, leading to unstable *in vivo* reprogramming efficacy (16). Third, post-injection protein corona formation reshapes EV surface properties, masks targeting peptides and accelerates hepatic clearance, drastically reducing lesion retention time in the same manner as synthetic nanoparticles. Fourth, long-term biosafety data remains insufficient; repeated high-dose EV infusion may trigger subtle peripheral inflammatory responses or splenic immune remodeling, with poorly characterized slow degradation kinetics in brain parenchyma. Finally, regulatory classification as advanced cell-derived medicinal products imposes stringent preclinical toxicology, biodistribution and immunogenicity testing requirements, and unified global quality control benchmarks for stroke EV formulations have not yet been fully established (19).

To mitigate these limitations, hybrid combinatorial strategies have been explored: loading engineered EVs into injectable MMP-responsive hydrogel scaffolds achieves sustained local depot release within the infarct cavity over weeks, reducing repeated systemic administration and peripheral clearance losses. Dual-ligand membrane modification and anti-fouling polymer shielding can also attenuate protein corona adsorption and improve lesion specificity. As a natural biomimetic delivery toolbox complementary to synthetic nanocarriers, engineered EVs fill an important gap in the spatiotemporal immune reprogramming technical framework, balancing biocompatibility and spatial–temporal targeting performance for long-term post-stroke immune modulation.

### 5.3 Biomaterial scaffolds for long-term regulation

For sustained and localized immunomodulation that guides tissue repair over weeks to months, implantable or injectable biomaterial scaffolds offer a powerful platform. These scaffolds can be engineered to create a defined “immunomodulatory niche” within the infarction cavity or peri-infarct region, providing long-term, tunable release of therapeutic factors and influencing host cell behavior. A primary strategy involves the local construction of an immune microenvironment by loading immunomodulatory molecules—such as anti-inflammatory cytokines (e.g., IL-10, IL-4), pro-repair chemokines, or small molecule drugs—into biodegradable, injectable hydrogels or microporous annealed particle (MAP) scaffolds. Upon injection into the stroke cavity, these materials form a three-dimensional network that can sequester and provide controlled, sustained release of these factors, directly shaping the phenotype of infiltrating and resident immune cells. For instance, hydrogels releasing IL-4 can persistently polarize microglia/macrophages towards a pro-repair M2 phenotype, while the slow elution of stromal cell-derived factor-1α (SDF-1α) can guide the homing of endogenous neural progenitor cells to the site of injury. Beyond serving as simple depots, the scaffolds themselves can be designed with specific physical and chemical properties that passively influence immune cell behavior, synergizing with the released bioactive cues. Material stiffness (elastic modulus) is a critical parameter; softer matrices mimicking healthy brain tissue have been shown to promote a more anti-inflammatory microglial phenotype compared to stiffer materials. Topographical features at the micro- and nano-scale, such as fiber alignment or surface roughness, can direct immune cell migration and morphology. Surface charge also plays a role; for example, positively charged materials may enhance the adsorption of specific proteins that influence subsequent cell adhesion and activation. Sulfated chitosan-based nanocarriers, for example, not only deliver drugs but their polyanionic nature can interact with growth factors and cells to promote neurovascular remodeling (55). Furthermore, scaffolds can be designed to be multifunctional. A scaffold could simultaneously provide structural support to fill the cavity, release a timed sequence of drugs (e.g., an anti-inflammatory followed by a pro-angiogenic factor), and present adhesive peptides (e.g., RGD) to support cell attachment and infiltration. This integrated approach of combining sustained biochemical signaling with engineered physical cues from the biomaterial itself allows for a comprehensive and durable reprogramming of the local immune milieu. This shifts the focus from transient systemic drug administration to establishing a long-lasting, tissue-engineered therapeutic environment that actively guides the complex and prolonged process of post-stroke immune resolution, tissue repair, and functional recovery.

Despite the prominent strengths of multi-stimulus responsive nanoparticles and injectable biomaterial scaffolds for spatiotemporally precise immune reprogramming, a series of unresolved translational bottlenecks severely restrict their clinical transformation, which are rarely discussed in the original one-sided description of technical merits. First, most ligand-modified nanocarriers suffer from unsatisfactory *in vivo* targeting efficiency. Even Angiopep-2 or VCAM-1 conjugated particles can be trapped by off-target peripheral vascular endothelial cells, and only a tiny fraction of administered nanoparticles ultimately accumulates within the penumbra or infarct core, greatly reducing effective local drug concentration (51). Second, spontaneous protein corona formation immediately after intravenous injection reshapes nanoparticle surface chemistry, masks targeted peptide ligands, disrupts stimulus-responsive degradation motifs, and triggers rapid phagocytic clearance by hepatic Kupffer cells and splenic macrophages, drastically shortening blood circulation half-life and weakening lesion tropism (59). Third, scalable manufacturing and reproducibility remain major industrial obstacles. Complex biomimetic membrane coating, multi-layer sequential encapsulation and MMP-sensitive peptide conjugation require ultra-precise synthesis parameters; slight fluctuations in reaction temperature, pH or raw material purity lead to inconsistent particle size, zeta potential and release kinetics between batches, failing to meet standardized pharmaceutical production requirements (60). Fourth, long-term biosafety and biodegradation risks cannot be overlooked. While short-term acute toxicity is generally mild, persistent microscale residual fragments after scaffold degradation may trigger chronic low-grade neuroinflammation, and accumulated inorganic nanomaterials such as layered double hydroxides potentially induce oxidative damage to neural progenitor cells over months (59). Fifth, strict regulatory barriers delay clinical trial progression. Nanomedicines and implantable biomaterials belong to combination medical devices, demanding far more rigorous preclinical toxicokinetic, biodistribution and immunogenicity evaluation than small-molecule drugs; unified global quality standards for stroke-targeted nanotherapeutics have not yet been established, bringing high cost and long-cycle approval risks. To mitigate these limitations, optimized material design strategies have been proposed, including dual-ligand co-modification to elevate target specificity, anti-fouling hydrophilic polymer shielding to inhibit protein corona adsorption, standardized continuous synthesis pipelines to unify batch properties, and fully organic biodegradable backbones without inorganic mineral fillers to minimize long-term residual toxicity. These ameliorative approaches lay a foundation for narrowing the gap between laboratory nanoplatforms and clinical stroke therapeutics.

Collectively, intelligent, pathology-responsive nanocarriers and biomaterial scaffolds possess unique strengths to realize spatiotemporally selective payload release, yet they face multiple interwoven translational limitations including insufficient targeting yield, protein corona interference, uncontrollable systemic clearance, unstable batch production and unresolved long-term biosafety concerns as elaborated above. Balanced optimization of material surface chemistry, structural composition and manufacturing workflow is indispensable to overcome these hurdles and advance bench-to-bedside translation of immune reprogramming nanomedicines.

## 6 Challenges in clinical translation and future prospects

### 6.1 From laboratory to clinical translation: key challenges

A critical limitation of the original translational discussion was insufficient dissection of why numerous neuroprotective and immunomodulatory agents show robust preclinical efficacy yet consistently underperform in human trials, an issue rooted in several interconnected translational disconnects.

Patient heterogeneity stands as the primary contributing factor. Preclinical stroke research largely relies on young, genetically identical rodents with a single defined ischemic trigger, while human stroke patients present vastly variable clinical backgrounds. Advanced age alone reshapes macrophage function and systemic immune trafficking to blunt treatment response (3); coexisting conditions including hypertension, diabetes and prior brain injury further shift baseline neuroimmune signaling (7). Stroke etiology also differs widely between patients, ranging from large-vessel thrombosis to cardioembolism with hemorrhagic transformation, alongside sex-specific immune regulatory pathways that alter injury severity and recovery trajectories (5). When unselected mixed cohorts are enrolled in clinical trials, therapeutic benefits observed in responsive subgroups become statistically diluted, leading to neutral overall trial outcomes even for compounds with clear biological activity in relevant patient subsets.

A second major mismatch lies in inconsistent therapeutic windows between preclinical protocols and real-world clinical practice. Most rodent interventions are administered within minutes of stroke onset, targeting the hyperacute inflammatory cascade; in clinical settings, however, the majority of patients arrive far beyond this narrow window. Many immunomodulatory candidates lose meaningful efficacy once early innate immune activation has fully propagated across the penumbra (19). Existing preclinical dosing regimens also rarely replicate the extended, multi-week immune remodeling process seen in human subacute and chronic recovery, with fixed single-shot delivery unable to adapt to shifting inflammatory profiles over time.

Manufacturing and reproducibility gaps create another layer of translational failure, especially for nanomedicines and extracellular vesicle therapeutics. Small-batch lab synthesis permits flexible fine-tuning of particle size, cargo loading and surface decoration, while clinical-scale production demands rigid, uniform manufacturing standards. Minor fluctuations in reaction conditions generate batch-to-batch variation in pharmacokinetics, lesion targeting and off-target toxicity (59). Extracellular vesicle preparations carry extra hurdles of low production yield and heterogeneous cargo profiles, making it difficult to maintain consistent therapeutic potency across clinical lots.

Complex regulatory pathways further slow bench-to-bedside progress. Conventional small-molecule immunomodulators follow established drug evaluation frameworks, whereas nanocarriers, engineered vesicles and injectable hydrogels are classified as combination medical devices requiring extended testing for long-term biodegradation, systemic immunogenicity and organ distribution (59). No unified global regulatory guidelines have been finalized for stroke-targeted immune reprogramming products, leading to inconsistent review standards, inflated trial costs and lengthy approval timelines that discourage clinical advancement of promising preclinical candidates.

Finally, the lack of biomarker-guided patient stratification undermines trial statistical power. Without multimodal imaging, liquid biopsy or multi-omics readouts to quantify individual inflammatory burden, trial teams cannot prospectively identify patients most likely to respond to a given therapy. A treatment effective for patients with severe sustained microglial activation and ectopic B-cell aggregates will show no significant overall benefit when mixed with patients with mild, self-resolving inflammation (61). Single-cell and spatial transcriptomic tools now provide a pathway to build personalized immune atlases, enabling researchers to group participants by their baseline neuroimmune state and match them to stage-tailored reprogramming regimens (62).

Taken together, these overlapping barriers create a consistent pattern of preclinical promise failing to translate to human clinical benefit. Addressing this cycle requires integrated solutions: biomarker stratification to homogenize trial cohorts, delivery platforms designed to match extended human therapeutic timelines, standardized scalable manufacturing workflows, and harmonized global regulatory standards tailored to biomaterial and EV-based stroke therapeutics. These corrective approaches lay the groundwork for the personalized translational framework elaborated in the subsequent section.

### 6.2 Personalized and precision therapeutic pathways

Against the multiple translational barriers outlined above, patient stratification supported by multi-modal biomarkers serves as the core strategy to bridge the gap between standardized preclinical dosing and the diverse immune profiles seen in human stroke patients, establishing the fundamental framework for individualized spatiotemporal immune reprogramming.

#### 6.2.1 Emerging clinically translatable biomarker platforms

Realizing truly customized immune modulation hinges on accessible, dynamic biomarkers that capture a patient’s evolving neuroimmune landscape, which can guide clinicians to match delivery carriers and intervention timing to individual pathological features. Several classes of biomarker tools have matured rapidly in translational stroke research, each with distinct clinical strengths and practical limitations.

Multimodal neuroimaging acts as the most convenient bedside readout. TSPO-labeled PET-MRI quantifies the scope and intensity of activated microglia across infarct core and penumbra, while contrast-enhanced MRI tracks dynamic BBB leakage, directly supporting spatially targeted nanotherapy planning. Circulating serum cytokines including IL-6 and TGF-β offer low-cost repeated monitoring, yet they cannot distinguish central nervous system inflammation from peripheral immune disturbances. Extracellular vesicle cargo signatures overcome this drawback: microglia-derived exosomes carry cell-specific microRNAs that faithfully reflect local brain immune shifts, serving as precise liquid biopsy indicators. Plasma proteomics and metabolomics further classify patients into pro-inflammatory high-risk or mild repair-prone subgroups by profiling immune adhesion molecules and glycolysis-related metabolites. Single-cell transcriptomics delivers the finest resolution to resolve rare pathogenic immune subsets, though high testing costs limit its routine clinical application.

No single biomarker type can fully reflect the whole post-stroke immune state. Integrated detection workflows combining TSPO neuroimaging, extracellular vesicle liquid biopsy and targeted metabolomic panels achieve a balanced evaluation of lesion spatial inflammation and systemic immune bias, laying a solid foundation for stratified individualized treatment. Beyond individual variations in age, comorbidities and genetic background, pathological heterogeneity of ischemic stroke itself profoundly reshapes the trajectory of neuroinflammation and determines therapeutic outcomes. Cortical and subcortical lesions trigger disparate immune activation patterns due to differences in vascular density and meningeal lymphatic distribution; large vessel occlusion induces severe, rapid hyperacute inflammation, while small vessel disease leads to slow, sustained low-grade immune disturbance. Reperfusion injury after mechanical thrombectomy introduces an extra wave of oxidative stress and myeloid infiltration, which demands timely early immune intervention, whereas cases complicated by hemorrhagic transformation exhibit exaggerated microglial activation and prolonged adaptive immune infiltration. All these subtype-specific immune shifts will change the ideal administration timing and expected efficacy of immune reprogramming approaches, which further highlights the value of multi-modal biomarkers to classify stroke subtypes and deliver customized therapeutic schedules.

#### 6.2.2 Multi-omics profiling and targeted precision intervention

To realize genuine precision stroke immunotherapy, clinical intervention strategies must combine real-time biomarker monitoring with multi-omic immune profiling. Multimodal imaging paired with liquid biopsies tracks dynamic inflammatory changes in real time, helping clinicians select the optimal therapeutic window for each patient. For instance, combined PET-MRI and peripheral immune testing generates a full picture of central and peripheral inflammation, and early hyperacute administration of DGMI delivers superior neuroprotective effects by balancing multiple inflammatory signaling pathways (61, 63).

Multi-omic profiling, particularly single-cell RNA sequencing, further deepens the granularity of immune evaluation. High-throughput sequencing identifies patient-specific microglial, monocyte and T cell subpopulations with divergent injury or repair functions (62). Epigenetic markers such as m6A RNA methylation also provide actionable clues to reshape the neuroimmune microenvironment (64). These high-resolution molecular insights shift therapeutic logic away from crude universal immunosuppression. Instead, tailored regimens can be designed to suppress pathological immune subsets while retaining endogenous tissue-repairing immune activity.

### 6.3 Cross-paradigm integration and future directions

**Figure 4:**
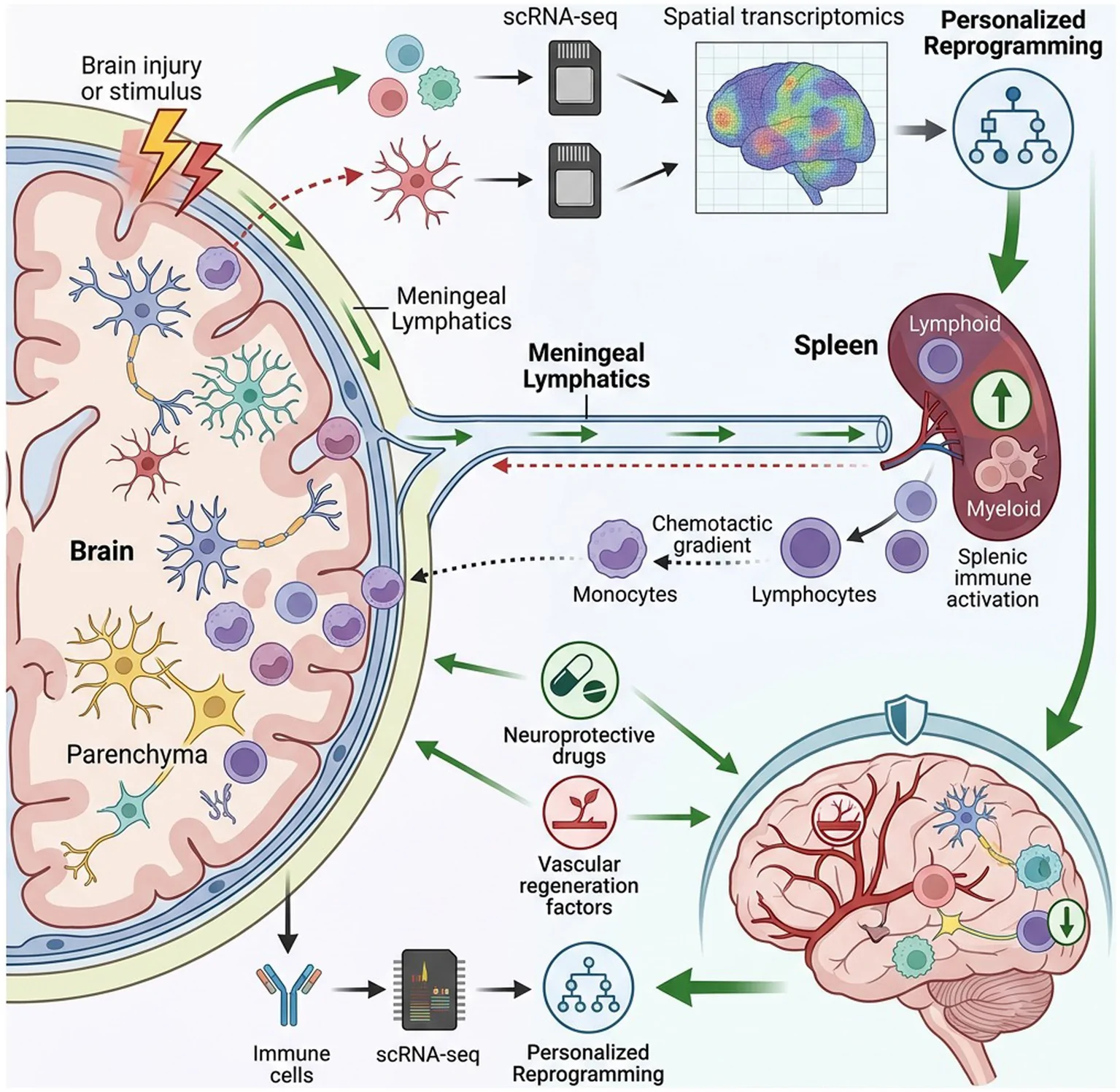
Integrated systems view/future paradigm. Integrated systemic view of brain-peripheral immune crosstalk and personalized immune reprogramming strategies. Brain-derived antigens and inflammatory mediators drain through meningeal lymphatics to spleen and peripheral lymphoid organs, reshaping systemic myeloid and lymphocyte pools. Single-cell RNA sequencing (scRNA-seq) and spatial transcriptomics generate patient-specific immune atlases to guide stratified personalized therapy, combining neuroprotective agents and vascular regeneration factors to remodel the immune microenvironment.

The future of spatiotemporal immune reprogramming for stroke lies in cross-paradigm integration and expansion to other neurological disorders. A key direction is the integration of intracerebral immune reprogramming with modulation of the brain-peripheral immune axis. Stroke is a systemic disease that disrupts peripheral immune organs like the spleen and alters immune cell trafficking. Future strategies should combine focal brain-targeted interventions with systemic approaches, such as modulating meningeal lymphatic drainage to clear brain debris or regulating splenic immune responses to prevent maladaptive peripheral immunosuppression and subsequent infections (30) (Figure 4). For example, remote ischemic conditioning has been shown to confer protection partly through immune modulation, influencing the expression of inflammatory genes in peripheral immune cells (65). Furthermore, combination therapy strategies will be essential. Immune reprogramming should be synergistically combined with neuroprotective agents, vascular regeneration promoters, and neuroregenerative therapies to construct a multi-pronged comprehensive repair regimen. This mirrors successful approaches in oncology, where stromal remodeling (e.g., reprogramming cancer-associated fibroblasts) is combined with immune checkpoint inhibition or immunogenic cell death induction to overcome therapeutic resistance (60, 66). In stroke, combining immune modulators [e.g., targeting IL-6 trans-signaling (67) or PD-L1 (68)] with agents that protect the blood–brain barrier or promote axonal sprouting could enhance recovery. Finally, the principles and technologies developed for stroke, particularly those involving nano-immunomodulation for precise spatiotemporal control of macrophage/microglia polarization (59), hold significant promise for application to other neurological conditions characterized by neuroimmune dysregulation. This includes traumatic brain injury, Alzheimer’s disease, and multiple sclerosis. In Alzheimer’s disease, for instance, lessons from managing amyloid-related imaging abnormalities (ARIA) associated with anti-amyloid immunotherapies are directly relevant to stroke care, highlighting the intricate link between vascular integrity and immune responses (69). By adapting immune reprogramming platforms to target disease-specific neuroinflammatory cascades, this therapeutic paradigm can be broadly translated to mitigate neuroinflammation and promote repair across a spectrum of central nervous system disorders.

As discussed above, stroke induces bidirectional brain-peripheral immune crosstalk mediated by meningeal lymphatic drainage and splenic immune remodeling. Future spatiotemporally precise immune reprogramming research should no longer focus solely on intracranial innate myeloid cells, but design combinatorial regimens that synchronously modulate central microglial/macrophage states, adaptive T/NK cell infiltration and peripheral splenic immune output, to break the long-standing research bias toward isolated central innate immunity (70, 71).

## 7 Conclusion

This review elaborates the original framework of spatiotemporally precise immune reprogramming, which outperforms three mainstream stroke immunotherapeutic approaches (stage-based intervention, personalized immunomodulation, single-target precision therapy) via three core breakthroughs. First, it unifies infarct core–penumbra spatial differences and multi-stage immune dynamic patterns into a coordinated therapeutic system, breaking the single-dimensional defect of time-only intervention strategies. Second, it abandons crude systemic anti-inflammation and advocates directional remodeling of repair-prone immune phenotypes, reconciling the contradiction between curbing acute brain damage and retaining intrinsic tissue-repair machinery. Third, it constructs a seamless bench-to-clinic translational chain by linking neuroimmune molecular mechanisms with stimuli-responsive nanocarriers and injectable biomaterial scaffolds—an integrated workflow unattainable through isolated biomarker screening or single-pathway targeting research. Collectively, these innovations fill critical gaps in existing therapeutic systems that fail to coordinate spatial targeting and timed delivery, laying a novel theoretical roadmap for subsequent stroke neuroimmune research.

Ischemic stroke immunotherapy is currently experiencing a fundamental conceptual shift away from universal immunosuppression toward lesion-localized, time-resolved immune remodeling. This paradigm transformation arises from updated recognition of inflammation’s dual harmful and regenerative properties, alongside comprehensive re-evaluation of the drawbacks inherent to traditional treatment logic. Rather than incremental optimization of existing agents, this shift marks a mature advancement in stroke immunology research: neuroinflammation is no longer treated as a pathological event to be fully eliminated, but as a time-variant biological cascade requiring delicate, staged regulation. Two complementary pillars underpin this innovative therapeutic model. The first is a systematic cellular target atlas that distinguishes damaging versus reparative activation states of microglia, monocytes, neutrophils and lymphocytes across distinct disease phases. The second consists of bioengineered precision delivery tools, including stimulus-sensitive nanoparticles, biomimetic membrane vehicles and sustained-release hydrogel reservoirs. These carriers penetrate biological barriers, selectively accumulate in injured cerebral zones, and release small-molecule, protein or gene payloads upon sensing local pH, enzyme and ROS signals. The core objective—targeted immune remodeling at matching lesion locations and pathological windows—can only be realized through deep integration of immunological mechanisms and material engineering.

Accumulated, sometimes contradictory preclinical data demands balanced therapeutic reasoning. Early studies predominantly highlighted inflammatory neurotoxicity and favored broad immunosuppression, while emerging evidence confirms indispensable immune functions such as cellular debris clearance, vascular regeneration and neurogenesis. Hence, modern therapeutic logic prioritizes recalibrating inflammatory balance rather than complete suppression. Practical intervention schemes can be designed to restrain hyperacute cytotoxic immune activity while amplifying repair-associated immunity in subacute stages, or to block pathological axes like the NLRP3 inflammasome without interfering with protective TGF-β signaling. Sequential-release multi-target nanoplatforms represent a viable technical route to reconstruct endogenous inflammation-resolving circuits.

If successfully translated into clinical practice, spatiotemporal immune reprogramming will deliver revolutionary stroke treatment benefits. Unlike recanalization therapies limited by ultra-narrow treatment windows, this strategy enables sustained penumbral protection and long-term modulation of subacute/chronic regenerative processes, ultimately reshaping the brain microenvironment to favor neural repair and improve permanent motor and cognitive deficits. Multiple unresolved translational barriers still stand in the way of clinical application, however: scalable standardized production of complex nanomedicines, long-term biosafety and biodegradation assessment, and wide inter-individual variation driven by stroke subtype, age, comorbidities and baseline immune profiles. Future research priorities include refining responsive delivery carriers to boost lesion specificity and tunable pharmacokinetic performance. Incorporating multimodal imaging, genomic and proteomic biomarkers will support patient stratification based on individual immune signatures, enabling customized reprogramming regimens matched to each patient’s pathological state. Rigorous, large-scale preclinical validation and stratified clinical trials are also necessary to confirm safety and identify patient subgroups most responsive to this novel therapeutic paradigm.
